# The Transcription Map of Human Papillomavirus Type 18 during Genome Replication in U2OS Cells

**DOI:** 10.1371/journal.pone.0116151

**Published:** 2014-12-30

**Authors:** Mart Toots, Andres Männik, Gaily Kivi, Mart Ustav, Ene Ustav, Mart Ustav

**Affiliations:** 1 Institute of Technology, University of Tartu, Tartu, Tartumaa, Estonia; 2 Icosagen Cell Factory OÜ, Ülenurme vald, Tartumaa, Estonia; 3 Estonian Biocentre, Tartu, Tartumaa, Estonia; 4 Estonian Academy of Sciences, Tallinn, Harjumaa, Estonia; National Institute of Health - National Cancer Institute, United States of America

## Abstract

The human osteosarcoma cell line U2OS is useful for studying genome replication of human papillomavirus (HPVs) subtypes that belong to different phylogenetic genera. In this study, we defined the HPV18 transcription map in U2OS cells during transient replication, stable maintenance and vegetative amplification by identifying viral promoter regions, transcription polyadenylation and splicing sites during HPV18 genome replication. Mapping of the HPV18 transcription start sites in U2OS cells revealed five distinct promoter regions (P_102_, P_520_, P_811_, P_1193_ and P_3000_). With the exception of P_3000_, all of these regions have been previously identified during productive HPV18 infection. Collectively, the data suggest that U2OS cells are suitable for studying the replication and transcription properties of HPVs and to serve as a platform for conducting high-throughput drug screens to identify HPV replication inhibitors. In addition, we have identified mRNA species that are initiated from the promoter region P_3000_, which can encode two E2C regulator proteins that contain only the C-terminal hinge and DNA-binding and dimerization domains of E2. We show that these proteins regulate the initial amplification of HPV18 by modulating viral transcription. Moreover, we show that one of these proteins can act as a transcriptional activator of promoter P_102_.

## Introduction

Human papillomaviruses (HPVs) are small DNA viruses that infect keratinocytes in the basal layers of mucosal or cutaneous epithelia. The more than 120 subtypes of HPV can be grouped phylogenetically into different genera (such as α-, β- and γ-HPVs) [1]. All HPVs have an 8 kb circular genome and similar genomic organization. The genome can be divided into early and late regions. The early region is primarily composed of genes that encode proteins that function in viral replication (E1, E2), transcription (E2) and the modulation of cellular functions (E4, E5, E6, E7). The late region encodes two capsid proteins, L1 and L2. These two regions are connected by the Long Control Region (LCR), which serves as the viral origin of replication and contains cis-elements for the regulation of viral transcription and genome maintenance, reviewed in [2]. The HPV replication cycle is dependent upon the differentiation program of the infected keratinocytes. Generally, the HPV replication cycle can be divided into three stages according to the mode of replication of the viral genome as an extrachromosomal genetic element (episome): (i) initial amplification of the HPV genome in the basal layer of proliferative keratinocytes, during which the viral copy number is increased to 50–100 genomes per infected cell; (ii) stable maintenance replication of the viral genome in the infected basal cells, which involves the segregation of the genome into the divided daughter cells; and (iii) final amplification of the HPV genomic DNA in the differentiating non-dividing keratinocytes, which is associated with late gene expression and assembly of the viral particles in the nucleus of the cell, reviewed in [3].

The most important and best-characterized HPVs are high-risk α-HPVs that infect mucosal cells and induce benign tumors that may progress to malignant hyperproliferative lesions in the mucosal epithelia of the vagina, cervix, anus and penis. In some cases, HPV causes cancers of the tongue, tonsils and neck. The high-risk HPV types 16, 18, 31, 33, 35, 39, 45, 51, 52, 56, 58 and 59 have been classified as group 1 carcinogens by the International Agency for Research on Cancer (IARC) [4]. Low-risk α-HPVs are associated with benign medical conditions such as condylomas, warts and laryngeal papillomatosis and, to some extent, with head and neck cancers. Cutaneous β-HPVs are associated not only with benign lesions, which are very common in the human population worldwide, but also with non-melanoma skin cancers [5], [6], [7]. Two preventive vaccines relying on reconstituted virus-like particles from expressed and purified L1 proteins targeting HPV6, HPV11, HPV16 and HPV18 (Gardasil) and HPV16 and HPV18 (Cevarix) have been developed and are reviewed in [8]. These vaccines are increasingly used in the human population to prevent infection by these viruses. However, these vaccines are ineffective at the elimination of established infections. Therefore, there is a clearly unmet medical need for drugs targeting the entirety of HPV replication during latent infections.

The development of effective anti-HPV drugs has been hampered by the limited availability of appropriate cell-based assay systems for screening for HPV replication inhibitors, as most human cell lines cannot support HPV genome replication. Cell lines established from mild dysplasias are known to be capable of stably maintaining high-risk HPV genomes as extrachromosomal genetic elements, albeit with a tendency toward spontaneous loss of the episomal genome, and to permit HPV genome amplification and packaging when grown in organotypic cultures. Among these cell lines, the HPV16-containing cell line W12 is the most studied [9], and references therein. In addition, raft culture and xenograft models have been developed for HPV studies [10], [11], [12]. All of these models can be used in studies of high-risk α-HPVs. In addition, isolated primary keratinocytes that maintain HPV genomic DNA and organotypic raft cultures that are based on these model systems can also be used [13], [14], [15], [16]. Recently, we demonstrated that the human cell line U2OS, which is derived from a moderately differentiated osteosarcoma, can support high- and low-risk and cutaneous HPV genome replication. The use of this cell line in HPV DNA replication studies is cost-effective and efficient [17]. In HPV replication studies, the U2OS cellular assay system has several advantages over keratinocyte-based systems for cell-based assays designed to identify potential inhibitors of all stages of HPV genome replication. In particular, U2OS cells support the replication of both oncogenic high-risk α-HPVs and low-risk HPVs and β-HPVs, permit the modeling of different stages of viral replication, are immortalized and can be cultivated in conditions that are suitable for automated high-throughput screening.

The reason why the human osteosarcoma cell line is capable of supporting the early stages of infection and latent HPV genome replication remains unclear. Genetic analysis suggests that the E1 and E2 proteins are necessary and sufficient to support HPV genome replication in U2OS cells [17]. However, the similarities and differences between the transcription maps of the HPV genome and of the virus in keratinocytes are not known. Recently, the full transcription map of HPV18 during productive viral infection in an HPV18-infected raft culture derived from human keratinocytes was published and has become a reference for HPV18 gene expression [18]. HPV18 is a high-risk α-HPV and the second most important oncogenic HPV type; this type is known to cause anogenital cancers [19]. Wang et al. mapped the HPV18 transcription start sites (TSSs) that clustered primarily in the 3′ end of the LCR and into the E7 open reading frame (ORF) of the viral genome. These researchers also identified the HPV18 polyadenylation cleavage sites (PASs) for early and late transcripts in the L2 ORF and LCR, respectively. In addition, the researchers constructed an HPV18 transcription map that includes several transcripts generated from five splice donor sites and six splice acceptor sites [18].

Herein, we present the results of a study of the HPV18 transcriptome during the different phases of viral genome replication in U2OS cells. We used 5′ and 3′ RACE assays to identify the viral promoter regions, transcription PASs and splicing sites in the viral early region. Based on the sequences of the RACE clones, we constructed a transcription map of the HPV18 early region in U2OS cells that reveals the remarkable similarities between HPV18 transcription regulation in U2OS cells and in productively infected raft cultures. In both systems, the same promoter regions and PASs were used. Similar to the raft culture transcription map, the HPV18 transcription map in U2OS cells is rather complex and consists of more than 20 different transcripts that initiate from five promoter regions and are generated from combinations of the five donor sites and five acceptor sites for splicing. Most of the viral transcripts detected in U2OS cells and described herein are identical in structure to those described previously for productive infection in raft cultures. In addition to more extensively studied P_102_, P_520_, P_811_, P_1193_, we identified a promoter region (P_3000_, located in the E2 ORF) in the HPV18 genome that has been reported once for HPV18 so far [20]. We show that this promoter could be used to produce HPV18 regulator proteins that contain only the C-terminal part of the E2 protein (E2C). We show that one of these proteins can act as a repressor or activator of transcription depending on the level of expression and therefore could modulate HPV18 genome replication in U2OS cells.

## Materials and Methods

### Cell lines and transfection

U2OS cells, which were obtained from the American Type Culture Collection (ATCC no: HTB-96), and the HPV18-positive U2OS subclone #1.13 [17] were grown in Iscove’s modified Dulbecco’s medium (IMDM) supplemented with 10% fetal calf serum (FCS). U2OS cells were transfected with the indicated amounts of HPV18 miniplasmid by electroporation (220 V, 975 µF) with a Bio-Rad Gene Pulser II that was supplied with a capacitance extender (Bio-Rad Laboratories). To induce episomal genome amplification (multiple times per cell cycle replication) in subclone #1.13, the cells were maintained in confluent conditions for 10–11 days without passaging and were fed with fresh medium every other day.

### Plasmids

**Figure 1:**
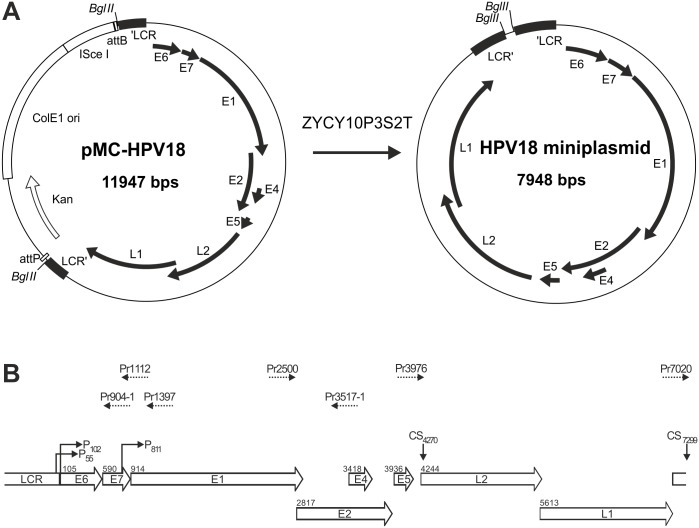
(A) Schematic maps of the parental plasmid pMC-HPV18 and the HPV18 miniplasmid produced from pMC-HPV18 in *E. coli* ZYCY10P3S2T according to the method described in ref. 22. (B) Schematic diagram of the HPV18 genome indicating the LCR and ORFs. The first nucleotides of the start codons for each ORF are indicated. The binding positions of the primers used for the 5′ and 3′ RACE assays in U2OS cells are shown above as dashed arrows. The numbers in the primer names indicate the binding positions (5′ end) on the HPV18 genome. Primer Pr3976 was described previously (ref. 44). The viral promoters P_55_, P_102_ and P_811_ and the transcription polyadenylation cleavage sites (CS_4270_ and CS_7299_) that were mapped during productive HPV18 infection are indicated (44).

The parental plasmid pMC-HPV18 was constructed for the production of HPV18 genome miniplasmids. A recognition site for the BglII restriction endonuclease was introduced into the HPV18 genome between nt 7473 and nt 7474 (herein, the numbering of the HPV18 genome is according to the NCBI Reference Sequence NC_001357.1). The same sites were used previously to insert loxP without observing changes in the patterns of early and late gene expression compared to unaltered HPV18 [21]. The modified HPV18 genome was linearized at the introduced BglII site and was cloned into the minicircle production plasmid pMC.BESBX [22], which had been linearized with BglII. The pMC backbone derived from pMC.BESBX permits the production of the HPV18 genome from pMC-HPV18 as a supercoiled minicircle that contains 92 bp of additional non-HPV sequences (Fig. 1A). Miniplasmid production was performed in *E. coli* strain ZYCY10P3S2T according to a previously published protocol [22]. Finally, the HPV18 genomes were purified from *E. coli* as supercoiled minicircles with the QIAfilter Plasmid kit (Qiagen). Mutations in the E8 and/or E2 ORFs were introduced by gene synthesis. The parental plasmid pMC-HPV18-E8^−^ (for the production of HPV18 miniplasmids that do not express the E8^∧^E2C repressor) was constructed by replacing the ATG start codon of the E8 ORF with the Thr codon ACG, as described in ref. [23]. The parental plasmid pMC-HPV18-E2C-1^−^ (for the production of HPV18 miniplasmids with mutations in the ATG start codon of the putative E2C-1 protein) was constructed by substituting a T nucleotide with C at position 3254. As a result, the putative ATG start codon of the E2C-1 protein was mutated to a Thr codon (ACG). The parental plasmid pMC-HPV18-E2C-2^−^ (for the production of HPV18 miniplasmids with mutations in the ATG start codon of the putative E2C-2 protein) was constructed by substituting an A nucleotide with G at position 3426. As a result, the putative ATG start codon of E2C-2 protein was mutated to a Val codon (GTG). The parental plasmid pMC-HPV18-2E2C (for the production of HPV18 miniplasmids with mutations in the ATG start codons of both putative E2C proteins) was constructed by substituting a T nucleotide with C at position 3253 and an A nucleotide with G at position 3426. As a result, the putative ATG start codons of both E2C-1 and E2C-2 proteins were mutated. The parental plasmids pMC-HPV18-E8-/E2C-2^−^ (for the production of HPV18 miniplasmids that express neither E8^∧^E2 nor E2C-2) and pMC-HPV18-E8-/E2C-1^−^ (for the production of HPV18 miniplasmids that express neither E8^∧^E2 nor E2C-1) were engineered by combining the above-described start codon mutations of the E8 ORF and the putative E2C proteins into the same viral genome. The parental plasmids pMC-HPV18-E8-/2-E2C^−^ (for the production of HPV18 miniplasmids that express neither E8^∧^E2, E2C-1 nor E2C-2) was engineered similarly to double mutants from parental plasmid pMC-HPV18-2E2C. Reporter plasmids pGL-18URR-Luc+, pGL-18 E2BS 3&4 and pRL-Tk were kind gifts from Reet Kurg, described in [23].

### RNA extraction and rapid amplification of cDNA ends (RACE)

PolyA^+^ RNA templates were extracted from U2OS cells that had been transfected with 500 ng of the HPV18 genome miniplasmid or from the U2OS subclone #1.13 with the Micro-FastTrack™ 2.0 kit (Invitrogen). Thereafter, 275 ng or 350 ng of the polyA^+^ RNA was used as a template for 5′ or 3′ RACE, respectively. Both the 5′ and 3′ RACE assays were performed with the SMARTer RACE cDNA Amplification Kit (Clontech) according to the manufacturer's instructions. The positions of the HPV18-specific primers that were used for the amplification of the RACE products are shown in Fig. 1B, and the sequences of the primers are listed in Table 1. The amplified RACE products were purified from agarose gels and then cloned and fully sequenced as single clones.

**Table 1:** Sequences of primers utilized for 5′ and 3′ RACE analyses.

Name	Sequence 5′-3′
Pr904-1	CTGCTGGGATGCACACCACGGACACAC
Pr1112	GTGCTGTCTCTAGCTCTGCCTGTTCAC
Pr1397	CACTACATACATTGCCGCCATGTTCGC
Pr2500	TGATGCAACGACCACGTGTTGGACATAC
Pr3517-1	ACGGACACGGTGCTGGAATACGGTGAG
Pr3976^1^	TGTATGTGTGCTGCCATGTCC
Pr7020	TTGGTTCAGGCTGGATTGCGTCGCAAG

1 Published in ref. 18.

### Southern blot analysis

Southern blot analysis to detect transient HPV18 replication was performed as described in ref. [17]. Briefly, low molecular weight DNA was extracted from U2OS cells that had been transfected with 500 ng of the HPV18 miniplasmid at 22, 46 and 71 hours post-transfection. The samples were linearized by digestion with BglI (Thermo Scientific), and the bacterially produced input DNA was fragmented by digestion with DpnI (Thermo Scientific). The purified and digested DNA was resolved on a 0.8% TAE-agarose gel, denatured and transferred to a Hybond-N^+^ filter (Amersham Pharmacia Biotech). Next, the DNA on the filter was hybridized with an HPV18-specific probe that was radiolabeled with α-^32^P-dCTP. Labeled DNA bands were detected via autoradiography by exposing the hybridized filter to x-ray film (AGFA).

### HPV18 copy number quantitation

The viral genome copy number in U2OS cells during replication was analyzed by quantitative real-time PCR (qPCR). Genomic DNA was extracted from U2OS cells that had been transfected with 2 µg of the HPV18 miniplasmid (wild-type or mutant genome) at 3, 5 and 7 days post-transfection. The samples were linearized by digestion with BglI (Thermo Scientific), and the bacterially produced input DNA was fragmented by digestion with DpnI (Thermo Scientific). For each qPCR reaction, 10 ng of DNA was used, and the reactions were performed with EvaGreen qPCR Mix Rox (Solis BioDyne) according to the manufacturer’s protocol on a 7900 HT Fast Real-Time PCR System (Applied Biosystems). The HPV18 replication signal was amplified with the following oligonucleotides (300 nM of each per reaction): 5′-GCGCTTTGAGGATCCAAC-3′ (HPV18 nt 110–127) and 5′-GTTCCGTGCACAGATCAG-3′ (HPV18 nt 148–165, complement strand). The analysis was performed according to the comparative threshold cycle (ΔCt) method. The results were calculated from the PCR cycle number in which the HPV signal exceeded the threshold value (Ct_HPV_). The Ct_GAPDH_ was detected as a normalization standard from the GAPDH gene sequence in the U2OS genome with the following oligonucleotides (300 nM of each): 5′-TACTAGCGGTTTTACGGGCG-3′ and 5′-ACAGGAGGAGCAGAGAGCGA-3′. The relative value C_N_, which reflects the average viral genome copy number per cell, was calculated from the data with the formulas ΔCt = Ct_HPV_–Ct_GAPDH_ and C_N_ = 2^−ΔCt^.

### Measurement of transcription activation and repression by the E2 proteins

U2OS cells were transfected with 100 ng of URR-Luc+ or 100 ng of 18 E2BS 3&4 together with different concentrations of various E2 plasmids. A non-specific pRL-Tk reporter plasmid expressing Renilla luciferase (25 ng) was added for normalization. The cells were lysed, and the luciferase activities were measured with a Glomax 20/20 luminometer (Promega) using a Dual-Luciferase reporter assay system following the manufacturer’s protocol (Promega).

### Immunoprecipitation and western blotting

U2OS cells were transfected with 1 µg of the pQMN-18E2, pQMCF-1-18E8E2, pQMCF-1-18E2C1, and pQMCF-1-18E2C2 expression vectors. At 36 h hours post-transfection, the cells were detached with PBS containing 3 mM EDTA and counted with a Countess Cell Counter (Invitrogen). The cells were then collected by centrifugation (200 g for 5 minutes), resuspended in RIPA buffer containing 1X protease inhibitor cocktail (Roche) and sonicated. The lysate was incubated with 30 µl of streptavidin-coated magnetic beads (Dynabeads M250) for 2 hours. The beads were collected, and each lysate was transferred to a new tube with 2 µg of biotinylated chicken HPV-18E2C antibody that had been raised against the C-terminal domain of the HPV18 E2 protein and incubated overnight at 4°C end-over-end. The next day, 50 µl of streptavidin-coated magnetic beads were blocked in 3% BSA-PBS for 2 hours, washed with PBS, added to the lysate and incubated for 4 hours at 4°C. The beads were then collected and washed 3 times in RIPA buffer containing 1x protease inhibitor cocktail (Roche). A total of 10 µl of 1x Laemmli buffer with 1 mM DTT was added per 3*10ˇ6 cells. Western blotting was performed as described in [24].

### Primer extension assay

U2OS cells were transfected with 2 ug of HPV-18 minicircle and total RNA was extracted 72 h post transfection using TRI-Reagent lysis solution (Molecular Reasearch Inc.). An oligonucleotide Pr881 was used for the primer extension assay (5′ACACAAAGGACAGGGTGTTC3′). The oligonucleotide was end-labeled with (gamma-P32) Adenosine 5′triphosphate (ATP) (Hartmann Analytics GmBH) by T4PNK (Thermo Scientific). 50 µg of total RNA was annealed with the labeled Pr881 oligonucleotide at 58**°**C for 2 h and reverse transcription was performed using AMV-Reverse Transcriptase (Life Technologies). The samples were treated with 10 U of RNAase A (Thermo Scientific) after which samples were purified by phenol-chlorophorm extraction and ethanol precipitation. The samples were eluted after which formamide containing loading dye was added and then denatured at 100**°**C for 10 minutes. 50% of total volume was loaded directly on a 50 cm 6% denaturing-PAA sequencing gel. Gel was vacuum-dried and primer extension signals were obtained by phosphoimager scanning (GE Healthcare) and HPV-18 specific primer extension products were obtained and quantitated using Image Quant TL software (GE Healthcare).

## Results

### Mapping of HPV18 transcription start sites (TSSs) in U2OS cells

**Figure 2:**
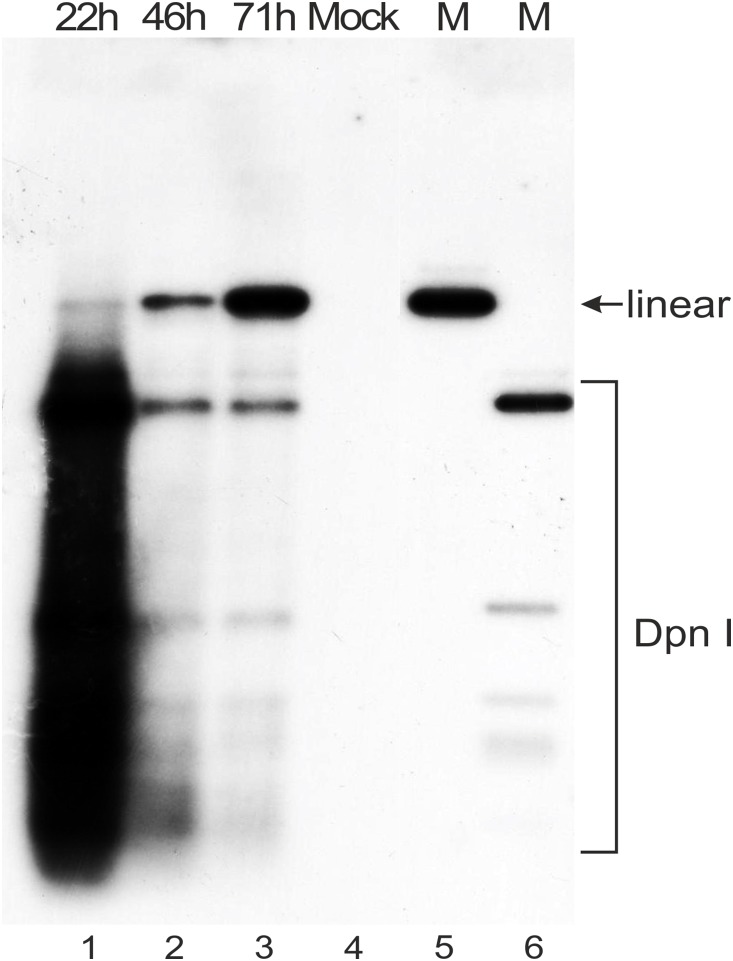
Southern blot analysis of HPV18 genome replication in U2OS cells that were transfected with 500 ng of the HPV18 genome miniplasmid. Extrachromosomal DNA samples were digested with BglI to linearize the HPV18 miniplasmid and with DpnI to fragment the bacterially produced input non-replicated plasmid. The samples were analyzed by Southern blotting after hybridization with an HPV18-specific radiolabeled probe. The DNA extraction timepoints (22, 46 and 71 hours) are indicated at the top. Extrachromosomal DNA extracted from mock-transfected U2OS cells was used as a negative control (lane 4). Size markers for the linearized HPV18 genome (lane 5, indicated by arrow) and for the DpnI+BglI digested fragments of the HPV18 genome miniplasmid DNA (lane 6) are included.

**Figure 3:**
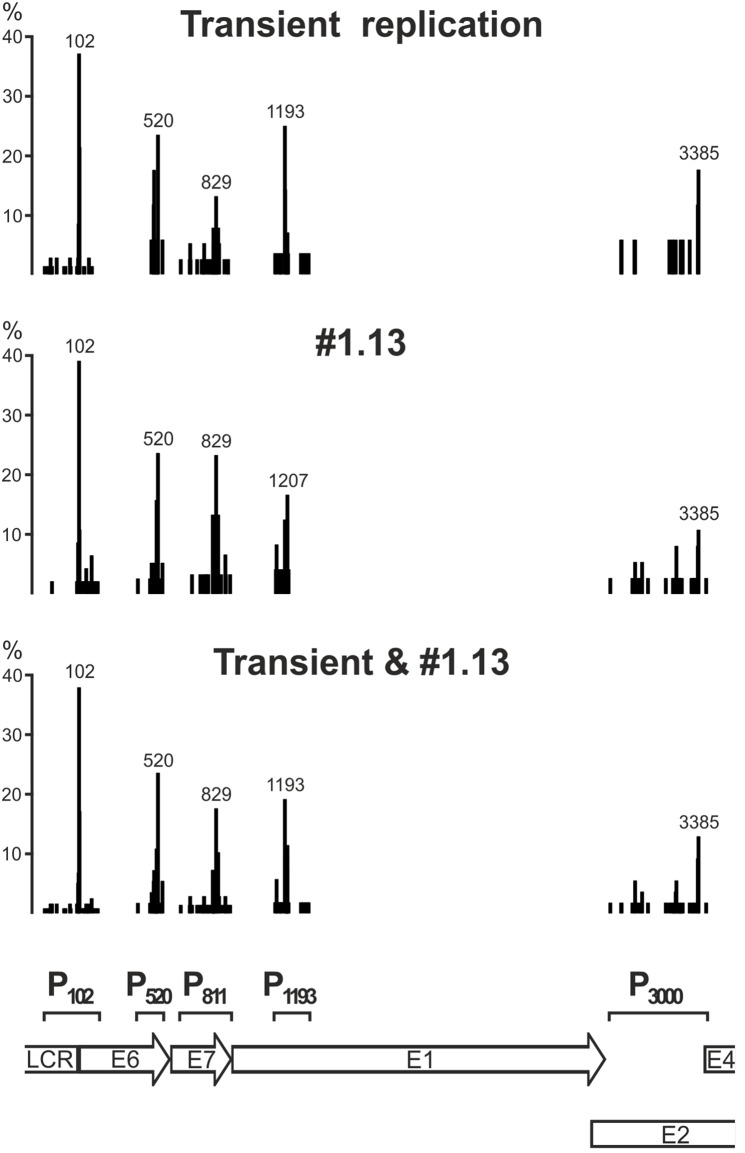
Promoter regions mapped by the clustering of TSSs in the early region of the HPV18 genome into five distinct promoter regions. The TSSs were identified by sequencing individual clones from 5′ RACE assays conducted on polyA^+^ RNA samples from U2OS cells that were transiently transfected with the HPV18 genome (169 clones from the 22- and 71-hour timepoints, upper diagram) and from U2OS subclone #1.13 cells (173 clones, middle diagram), which stably maintain HPV18 episomal DNA. In the bottom diagram, data from the transient transfection and #1.13 are summarized. Each bar in the diagrams represents a single TSS, and the height of each bar represents the percentage of mRNAs that initiated from that TSS in a particular promoter region. For each promoter region, the nt position of the most prevalent TSS is indicated above the highest bar. At the bottom of the figure, the locations of the defined promoter regions (P_102_, P_520_, P_811_, P_1193_ and P_3385_, indicated with square brackets) in the HPV18 genome are indicated.

**Figure 4:**
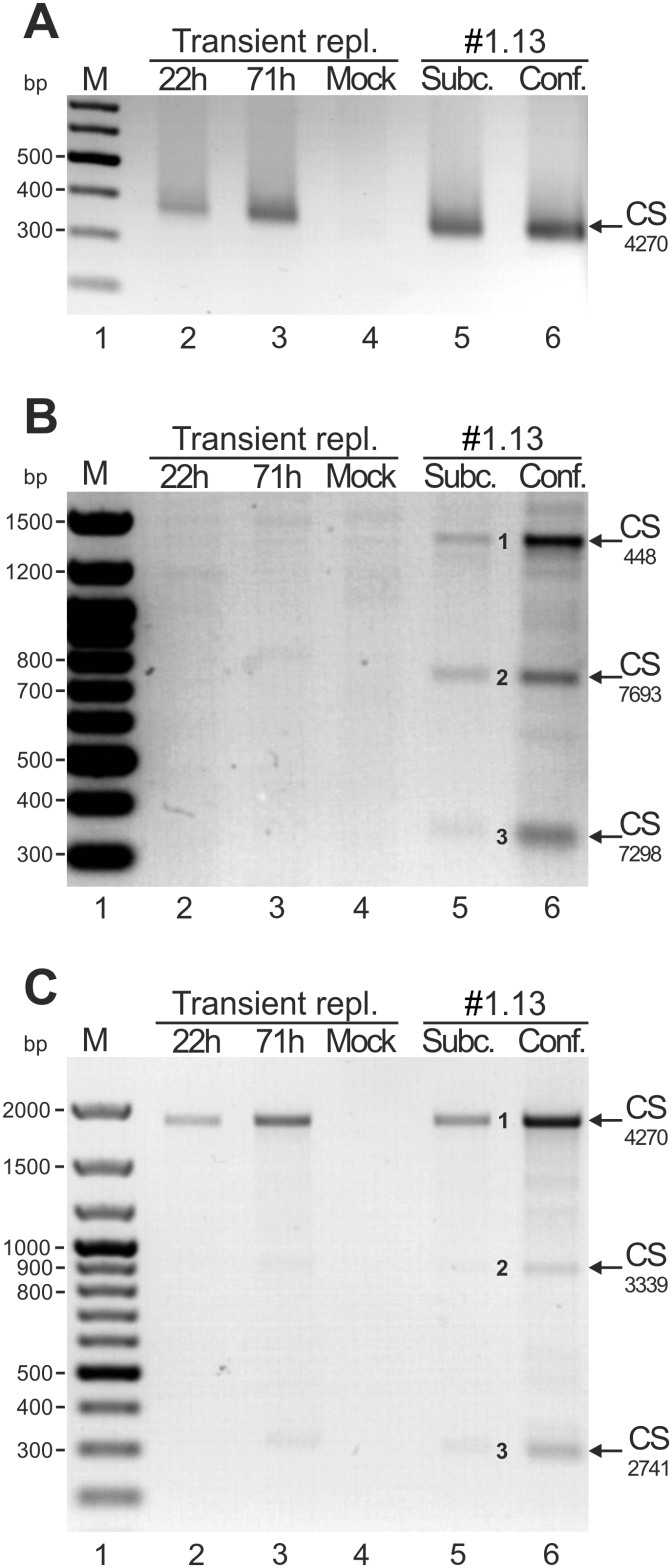
Mapping of the HPV18 transcription polyadenylation cleavage sites (CSs) by 3′ RACE. The assays were conducted in U2OS cells that were transiently transfected with the HPV18 genome (timepoints are indicated above each gel) and in HPV18-positive U2OS subclone #1.13 cells that were cultured in subconfluent or confluent conditions. (A) 3′ RACE analysis of the HPV18 early region CS with the HPV18-specific primer Pr3976. The 3′ RACE product (indicated by an arrow) represents the CS at 4270. (B) 3′ RACE analysis of the HPV 18 late region CS with the HPV18-specific primer Pr7020. Three 3′ RACE products (marked as 1, 2 and 3 and indicated by arrows) represent the CSs at nt 448, nt 7693 and nt 7298, respectively. (C) 3′ RACE analysis with the HPV18-specific primer Pr2500 for analysis of the CSs of E1-encoding transcripts. Three 3′ RACE products (marked as 1, 2 and 3 and indicated by arrows) represent the CSs at nt 4270, nt 3339 and nt 2741, respectively.

The 5′ ends of HPV18 transcripts in U2OS cells were mapped by 5′ RACE analyses of polyA^+^ RNA from U2OS cells that had been transfected with 500 ng of the HPV18 miniplasmid. RNA extracted from the 22 h and 71 h timepoints was used. Within this timeframe, HPV18 genome replication is initiated, and an increase in the genome copy number was clearly detected (Fig. 2). In addition, the HPV-positive U2OS subclone #1.13 [17], which contains the HPV18 genome as a stable multicopy episome, was used for parallel TSS mappings. The HPV18-specific primers Pr904-1, Pr1112, Pr1397 and Pr3517-1 (Fig. 1) were used to amplify the 5′ RACE products. The TSS data were collected from the sequences of a total of 342 clones of 5′ RACE products, which were derived from the HPV18 transient replication samples (169 clones) and the cell line #1.13 (173 clones). The results shown in Fig. 3 indicate the clustering of TSSs into five distinct promoter regions that are referred herein as P_102_, P_520_, P_811_, P_1193_ and P_3000_. This observed clustering pattern was very similar in the transient replication samples and in subclone #1.13 passaged at low density. In Fig. 3, bars represent the frequencies of the different TSSs in each promoter region as percentages of the representative 5′ RACE clones that were identified for each particular region, indicating that certain positions are used most frequently as TSSs, although neighboring sequences are also involved. These data suggest that the early region of the HPV18 genome carries five promoter regions (Fig. 3). In addition HPV18 genome harbors 7 polyadenylation cleavage sites (CSs; Fig. 4).

The promoter region P_102_ was defined in U2OS cells to extend from nt 7779 in the LCR to nt 201 in the 5′ end of the E6 ORF. This promoter has been described previously [25], and its activity has been demonstrated in cervical carcinoma cell lines [26], transfected primary human keratinocytes [27] and an HPV18-infected keratinocyte raft culture [18]. In U2OS cells, 38% of the analyzed clones that started from P_102_ had a TSS at nt 102, whereas 68% had a TSS at nt 105 (17%), 100 (7%) or 97 (5%). Approximately 10% of the TSSs in P_102_ were in the LCR region upstream of nt 55, which extended to nt 7779.

**Figure 5:**
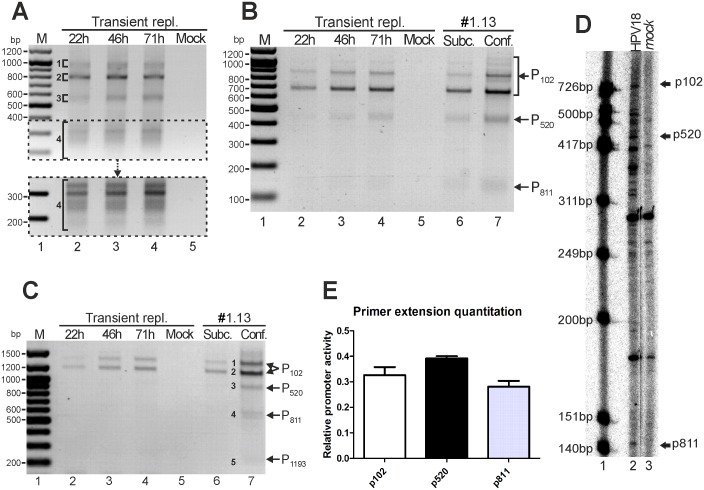
Mapping of the HPV18 splice junctions in U2OS cells by 5′ RACE. The assays were conducted with U2OS cells that had been transiently transfected with the HPV18 genome (timepoints indicated above each gel) and with HPV18-positive U2OS subclone #1.13 cells that were cultured in subconfluent or confluent conditions. (A) 5′ RACE analysis of HPV18 transcripts that were produced in U2OS cells during transient replication. An HPV18-specific primer (Pr3517-1) that binds to a site in the E4 ORF was used. All distinct products (regions marked as 1, 2, 3 and 4) were purified, cloned and sequenced. The natures of the represented transcripts are described in the results and are shown in Fig. 6. (B) 5′ RACE analysis of RNAs in U2OS cells with an HPV18-specific primer (Pr904-1) that binds to a site in the E7 ORF. The dominant RACE product (size ∼700 bp) represents the spliced transcripts as -233/416- (RNAs A, C and K in Fig. 6). Products representing the RNAs that initiated from promoters P_102_, P_520_ and P_811_ are indicated. (C) 5′ RACE analysis of E1-encoding mRNAs with an HPV18-specific primer (Pr1397-1) that binds to a site in the E1 ORF. The indicated products (1–5) were cloned and sequenced. Products representing the RNAs that initiated from promoters P_102_, P_520_, P_811_ and P_1193_ are indicated. (D) Primer extension assay of HPV18 genome transiently replicating in U2OS cells. Signals are obtained with HPV18-specific primer Pr881. Lane 1 serves as marker (ΦX174 DNA/HinfI; Promega), lane 2 is HPV18 genome and lane 3 is mock sample. Bands representing transcripts arisen from HPV18 promoters are indicated. (E) Primer extension assay quantitation of transiently replicating HPV18 in U2OS cells. Values indicate proportion of overall promotoral activity.

The promoter region P_520_ spans mainly from nt 479 to nt 544 in the E6 ORF; the TSS at nt 414 was detected in only one instance. This region was not previously well defined as a viral promoter; however, the detection of spliced transcripts that had initiated near nt 500 was reported in HPV18-infected raft cultures [18]. Of the analyzed clones, the most prevalent TSSs in this region were detected at nt 520 (24%), 514 (11%) and 500 (7%). During transient replication, the amounts of the P_520_-initiated transcripts increased over time (lanes 2–4, Fig. 5B). Increased activity levels were also detected in the HPV18-positive subclone #1.13 cells when maintained in confluent conditions to permit amplificational replication of the episomal HPV18 genome (lanes 6–7, Fig. 5B).

The promoter region P_811_ spans from nt 689 to 903 in the E7 ORF, although in one instance, a TSS was identified at nt 641. This promoter was described previously in HPV18-infected raft cultures and was defined as a late promoter [18]. In U2OS cells, the most prevalent TSSs initiated at nt 829 (18%, Fig. 2), 840 (10%), 811 (7%) and 814 (6%). In 5′ RACE analyses of HPV18 transient replication samples, the amounts of the products that initiated from P_811_ were relatively low in comparison to the amounts that initiated from the P_102_ and P_520_ regions (lanes 2–4, Fig. 5B). The relative amounts of transcripts were higher in subclone #1.13 cells during passaging, and the amount of P_811_-initiated transcripts increased considerably when the cells were maintained in confluent conditions (lane 7, Fig. 5B).

Due to the semi-quantitative nature of the 5′ RACE assay, primer extension quantitation (described in materials and methods section) was performed. Results in Fig. 5, panels D and E indicate that p520 showed slightly higher promotoral activity than p102 (39% vs 33%) and p811 promotoral activity was 28% of overall promoter activity.

The promoter region P_1193_ spans from nt 1142 to nt 1319 in the E1 ORF. Like P_520_, this region was not previously well defined as a promoter region, although a spliced transcript (1202-1357/3434) was reported in HPV18-infected raft cultures [18]. In U2OS cells, we determined that the prevalent TSSs initiated at nt 1193 (19%, Fig. 2), 1207 (12%), 1195 (10%) and 1205 (10%).

**Figure 6:**
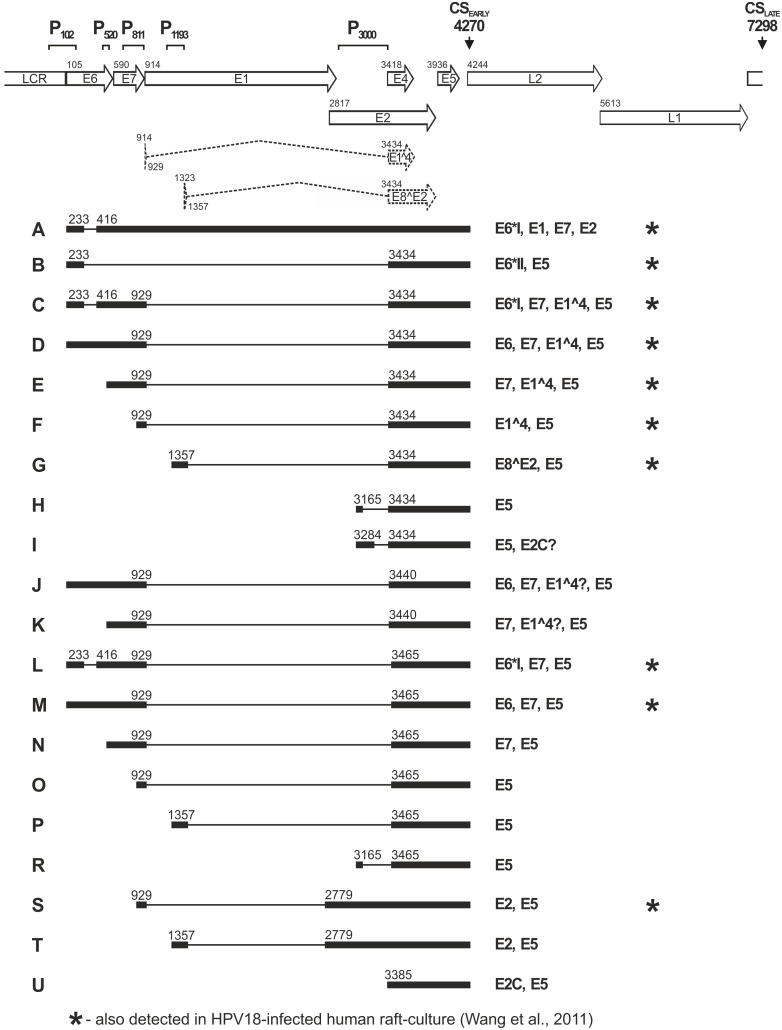
Summary of the HPV18 transcripts that were mapped in transiently transfected U2OS cells and in subclone #1.13 cells by 5′ RACE analyses with primers Pr1397 (RNA A) and Pr3517-1 (RNAs B-U). Top, a linear depiction of the HPV18 genome with the ORFs, LCR, E1^∧^E4 and E2^∧^E8 coding sequences spanned over two exons (dashed line), along with the promoter regions P_102_, P_520_, P_811_, P_1193_ and P_3385_ and the major polyadenylation cleavage sites CS 4270 and CS 7298. All transcripts depicted herein (designated with letters A-U, shown at left) are shown with exons (solid boxes) and introns (lines) and with the splicing donor and acceptor sites (nt numbers). The coding potential is described to the right of each transcript. The RNA species previously described in HPV18-infected raft cultures [18] are indicated by asterisks. In addition, the splicing pattern -3165/3434- (as for RNA H herein) was reported previously in both raft culture and clinical samples (41).

The promoter region P_3000_ extends over quite a large area in the E2 ORF (nt 2918–3426), similarly to the previous study conducted in vitro and in Hela cells [20]. We identified an unspliced transcript that initiated from the TSS at nt 2975–3426 as the major RNA product (RNA U, Fig. 6) from this promoter region, which encodes the E2 repressor protein (see below). In addition, spliced RNAs (H, I and R, Fig. 6) were detected.

Analyses of the nucleotide sequences around the TSSs revealed that for promoters P_102_, P_520_, P_811_ and P_1193_, transcription initiated mostly at the purine nucleotide A. Moreover, the surrounding nucleotides of the TSSs were frequently consistent with the Inr consensus sequence YYA_+1_NWYY in human cells [28], and references therein. In particular, a relatively high percentage of the analyzed clones of promoters P_102_, P_520_, P_811_ and P_1193_ had the TSS motif YA_+1_NW. The TSS motif data are summarized in Table 2.

**Table 2:** Frequency of human consensus transcription start site motifs in promoter regions.

TSS	P_102_ (n = 116)	P_520_ (n = 55)	P_811_ (n = 68)	P_1193_ (n = 52)	P_3385_ (n = 51)
**A_+1_**	102 (88%)	29 (53%)	39 (57%)	31 (60%)	23 (45%)
**YA_+1_NW**	59 (51%)	21 (38%)	29 (43%)	24 (46%)	5 (10%)

### Mapping of HPV18 transcription polyadenylation cleavage sites (CSs) in U2OS cells

The functional HPV18 polyadenylation sites (PASs) were mapped by identifying the polyadenylation cleavage sites (CSs) by 3′ RACE analyses of polyA^+^ RNA extracted from U2OS cells that had been transfected with the HPV18 genome miniplasmid (22 and 71 h timepoints; data shown in Fig. 2) and from the U2OS subclone #1.13. Previously, the CSs for the early and late transcripts in productively infected human keratinocytes were mapped to nt 4270 and nt ∼7300, respectively [18].

First, we conducted 3′ RACE analyses with the HPV18-specific primer Pr3976 (44; Fig. 1B) to determine whether the polyadenylation CS at nt 4270 was utilized during HPV18 replication in U2OS cells. In transient transfection samples and in subclone #1.13 cells that were maintained in either subconfluent or confluent conditions, a single ∼350 bp RACE product was observed that was not detected in mock-transfected U2OS cells (Fig. 4A). This product was cloned from all samples from transient transfections and #1.13 cells. Sequencing of the clones from all of the samples indicated a CS in the region between nt 4253 and nt 4272 that was located most frequently at nt 4270 (57% of the clones).

To analyze usage of the late PAS, 3′ RACE analyses were performed with the HPV18-specific primer Pr7020 (Fig. 1B). We were particularly interested in whether the maintenance of #1.13 cells in confluent conditions might to some extent reflect the events of the late-phase viral life cycle [17], which should be associated with an increased use of the late PAS. The 3′ RACE assay results are shown in Fig. 4B. No 3′ RACE products were observed in the transiently transfected samples (lanes 2–4), indicating that the late PAS with a CS at ∼nt 7300 was not used during transient replication of the HPV18 genome in U2OS cells. In contrast, three discrete major products with approximate sizes of 1400 bp, 750 bp and 350 bp (1, 2 and 3, respectively, Fig. 4B) were observed in subclone #1.13 cells (lanes 5 and 6), and the signal intensities of these products increased when the cells were maintained in confluent conditions (lane 6) compared to cells maintained in subconfluent conditions (lane 5). Sequencing of the cloned products revealed that product 3 (8 clones) represented the CS between nt 7288 and nt 7307 and occurred most often at nt 7298 (30% of clones). Thus, the cis-elements that were defined previously for the polyadenylation of viral late transcripts in productive infections were active in subclone #1.13 cells, particularly when the cells were maintained in conditions associated with viral genome amplification. Two larger products (1 and 2, Fig. 4B) were also cloned and sequenced from subclone #1.13 cells. Both of these products represented the more downstream CS and likely were generated from transcripts that passed through the CS located around the nt 7300. Product 2 represents the CS at nt 7692 or 7693. According to the analysis of the HPV18 genome, the putative polyadenylation signal motif AAUAAA was found at nt 7278, and a U/GU rich sequence was identified immediately downstream; both of these are essential cis-elements for RNA polyadenylation [29]. Product 1 represents the CS at nt 375–471, which occurred primarily at nt 448.

The possible existence of additional PASs in the HPV18 early region was analyzed using a 3′ RACE assay with a primer that binds to the E1 ORF of the HPV18 genome (Pr2500; Fig. 1B). This analysis was also performed to determine the PASs for the replication protein E1-encoding transcripts (RNA A, Fig. 6). A major RACE product with an approximate size of 1900 bp (indicated as 1, Fig. 4C) was observed in both U2OS cells that had been transfected with the HPV18 genome and subclone #1.13 cells. In addition, two minor products with approximate sizes of 900 bp and 300 bp (2 and 3, Fig. 4C) were observed at 71 h post-transient transfection and in the subclone #1.13 cells (lanes 3, 5 and 6). Again, the signal intensities of all subclone #1.13 products increased when the cells were maintained in confluent conditions compared to cells that were maintained in subconfluent conditions (lanes 6 and 5, respectively). Sequencing of the clones revealed that product 1 represented the early PAS that had been previously identified with primer Pr3976 with a CS at nt 4270. The minor products 2 and 3 represent polyadenylation CSs in the nt 3337–3357 region, primarily at nts 3339 and 3344, and in the nt 2738–2752 region, primarily at nt 2741, respectively. Thus, both transcripts, when used as the templates for products 1 and 2, can encode the full-length E1 protein. We also conducted an analysis of the HPV18 genome by a manual examination of the sequence with the support vector machine-based method PolyApred [30] to predict putative PASs that might precede the CSs at nt 3339 and 2741. However, no clear PAS motifs were identified.

### Mapping of HPV18 splice junctions and construction of the viral early transcription map in U2OS cells

The structures of viral transcripts that were expressed by HPV18 during genome replication in U2OS cells were determined by 5′ RACE analyses with the HPV18-specific primer Pr3517-1, which binds to a sequence in the E4 ORF (Fig. 1B). The 5′ RACE template polyA^+^ RNA was extracted from U2OS cells at 22 or 71 h after transfection. In this timeframe, the copy numbers of the replicated episomal HPV18 genome increased; thus, these samples represented the initial transient replication of the virus (Fig. 2). The 5′ RACE products, which were observed as a complex pattern, were qualitatively similar at all timepoints (Fig. 5A). In addition, a 5′ RACE assay with primer Pr3517-1 was performed with polyA^+^ RNA that was extracted from U2OS subclone #1.13, in which the HPV18 genome is maintained as a stable episome. We observed a very similar pattern of RACE products in all samples (data not shown). The data from 270 clones were used to construct a transcript map (Fig. 6 and specified below).

Our analysis revealed the number and location of splicing sites (splicing donor sites at nt 233, 416, 929 and 1357; splicing acceptor sites at nt 416, 2779, 3434 and 3465; Fig. 6), which had been described previously in productive HPV18 infections [18]. Product 2 in Fig. 5A represents the most intensely expressed product, a transcript initiated from P_102_ that was double-spliced as -233/416-929/3434- (RNA C, Fig. 6). In addition, approximately 25% of the clones (8 clones from 33) from product 1 (Fig. 5A) originated from the same RNA sequence with an alternate start site at the extreme 5′ end of P_102_ (nt 7779–7842). Notably, this transcript was previously mapped as an abundant transcript in HPV18-infected raft cultures (RNA C in [18]) that could encode the viral proteins E6*I, E7, E1^∧^E4 and E5 [18]. The RNA that initiated from P_102_ and was spliced as -233/416- was also abundant in subclone #1.13 (product marked by asterisk, Fig. 5B). Product 1 in Fig. 5A also represents the transcript that initiated from P_102_ and was spliced as -929/3434- (RNA D, Fig. 6). Two clones originated from RNAs that initiated from P_102_ and were spliced as -929/3465- and as -929/3440-. In addition, rare RNAs were observed that used the splicing donor site at nt 2779. These rare RNAs initiated from P_811_ and were spliced as -929/2779- (RNA S in Fig. 6) or initiated from P_1193_ and were spliced as -1357/2779- (RNA T, Fig. 6). Product 3 in Fig. 5A was generated primarily from RNA that initiated from the promoter P_520_ and was spliced as -929/3434- (RNA E in Fig. 6). A small fraction (<5%) of the clones from product 3 (Fig. 5A) represent similar transcripts with alternate splicing donor sites at nt 3440 or nt 3465 instead of nt 3434. The clones obtained from the designated product-containing region 4 (Fig. 5A) primarily yielded sequences representing RNAs that initiated from the promoters P_811_, P_1193_ and P_3000_ (RNAs F, G, I, O and P, Fig. 6). However, one of the 78 clones isolated from transiently transfected U2OS cells and two of the 86 clones isolated from subclone #1.13 originated from RNA that initiated from P_102_ and was spliced as -233/3434 (RNA B, Fig. 6). Most of the transcripts described herein and depicted in Fig. 6 were present in cells that were transiently transfected with HPV18, including those at the very early 22 h timepoint. Thus, these transcripts represent genuine viral transcripts and were not generated by integration events of the HPV18 genome in the #1.13 subclone. The exceptions are RNA L (105-233/416-929, 3465), which was represented by a single clone, and RNAs H and R, which initiated from P_3000_. All of these transcripts were detected only in subclone #1.13 cells that were maintained in conditions suitable for viral genome amplification. However, RNA L was described previously in HPV-18 infected raft cultures [18], and a splicing pattern for RNA H was identified previously in raft cultures and clinical samples [31].

The analysis of splicing acceptor site usage in the E2 ORF revealed a strong preference for the splicing donor site at nt 3434 over the sites at nt 3440 and nt 3465. In transient replication samples, 91% of the clones of transcripts that had been spliced at one of these three sites had used the splicing acceptor site at nt 3434, and only 8% and 1% of clones had used the sites at nt 3465 or 3440, respectively. A similar pattern was observed in subclone #1.13 cells (94% of clones used site nt 3434, 6% used site nt 3465, and 0% used site nt 3440). The early transcripts initiated from P_102_, P_520_ or P_811_ and spliced as -929/3434- can encode the E1^∧^E4 protein, and transcripts initiated from P_1193_ and spliced as -1357/3434 can encode the E8^∧^E2 regulator. The acceptor site at nt 3440 may be functionally equal to the site at nt 3434 because these sites are in the same reading-frame and only two amino acids (Pro-Val for E1^∧^E4 and Ser-Thr for E8^∧^E2) are deleted from the encoded protein if the site at nt 3440 is used instead of the site at nt 3434. However, the use of the splicing acceptor site at nt 3465 changes the reading frame for the protein.

### Identification of E1-encoding transcripts

The expected 5′ RACE products of Pr3517-1 representing E1-encoding mRNAs are more than 3 kb in size, which complicates the cloning or detection of these products. Thus, to analyze the transcripts that encode the E1 protein, 5′ RACE was performed with the HPV18-specific primer Pr1397, which binds to a sequence in the E1 ORF after the splicing donor site at nt 1357 (indicated in Fig. 1B). Two distinct RACE products (1 and 2, Fig. 5C) were observed at all transient replication timepoints and in subclone #1.13 cells. Sequencing analysis revealed that both products represent transcripts that initiated from promoter P_102_. The prevalent product 2 represented the transcript that was spliced as -233/416- and included the coding sequences for the viral proteins E6*I, E1, E2, E7, E4 and E5 (RNA A, Fig. 6); this product was also identified in HPV-18-infected raft cultures [18]. Product 1 primarily represented unspliced transcripts that initiated from P_102_ and transcripts that were spliced as -233/416- but that initiated at the extreme 5′ end of the promoter (nt 7804–7818). However, three additional products, including the E1 ORF sequence, were observed only in subclone #1.13 cells that were maintained in confluent conditions to induce the HPV18 genome amplification more than once per cell cycle. These products represent amplification-specific E1 ORF transcripts (3, 4 and 5, Fig. 5C). Sequencing of products 3, 4 and 5 revealed a uniform representation of unspliced RNAs that initiated from promoters P_520_, P_811_ and P_1193_, respectively. Thus, in comparison to the stably maintained viral genome, transcription of E1 ORF sequence-containing mRNAs from all early promoter regions (except P_3000_) is activated during the amplification mode of HPV18 genome replication. The mRNAs that initiated from promoters P_102_, P_520_ and P_811_ are capable of encoding the E1 protein.

### The putative function of the mRNAs expressed from P_3000_

**Figure 7:**
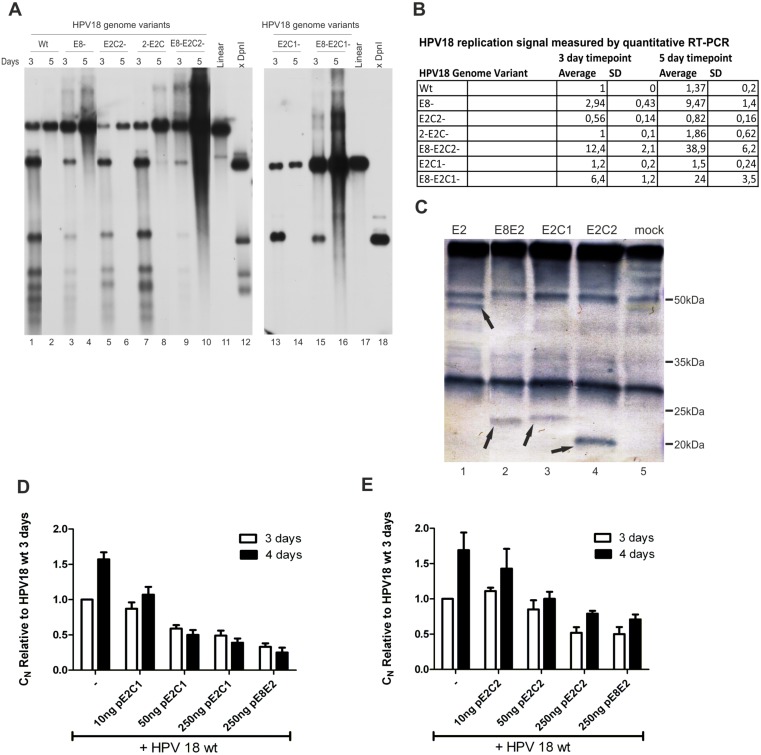
Mutational analysis of the functions of the putative E2C1 and E2C2 proteins expressed from promoter P3385. **A:** Southern blot analysis of the transient replication of different HPV18 genome mutants. U2OS cells were transfected with 2 µg of HPV18 *wt*, E8-, E2C2-, 2-E2C-, E8-E2C2-, E8-2-E2C-, E2C-1 or E8-E2C1- minicircles. Genomic DNA was extracted 3 and 5 days after the transfection, linearized with BglI and treated with DpnI to distinguish between transfected and replicated DNA. The samples were analyzed by Southern blotting after hybridization with an HPV18-specific radiolabeled probe. Size markers for linearized HPV18 (lanes 11 and 17) and for the DpnI-digested fragments of the HPV18 (lanes 12 and 18) are included **B:** U2OS cells were transfected with 2 µg of the indicated HPV18 genome mutants, and genomic DNA was extracted 3 and 5 days after the transfection. Samples were digested with BglI and DpnI, and the replication of different HPV18 genome mutants was measured by a qPCR-based analysis of the viral relative copy number (C_N_). The value obtained from the HPV18 *wt* 3-day time point was set to 1, and the C_N_ values of other samples are expressed relative to this point. The average and standard deviation (SD) of at least three independent experiments are shown. **C:** U2OS cells were transfected with the expression plasmids of HPV18 full-length E2, E8E2, E2C1 and E2C2. IP-Western Blot analyses was performed to evaluate the expression levels and MWs of different HPV18 E2 variants. Arrows indicate the positions of the full-length E2 (lane 1), E8^∧^E2 (lane 2), E2C1 (lane 3) and E2C2 (lane 4). Mock transfection is shown in lane 5. **D and E:** U2OS cells were transfected with 2 µg of HPV18 *wt* minicircle plasmid alone or together with different concentrations (10, 50 and 250 ng) of either the expression vector or the E2C-1 or E2C-2 proteins. The E8ˇE2 expression vector (250 ng) was added as a control. Genomic DNA was extracted 3 and 4 days after the transfection, linearized with BglI and treated with DpnI. A qPCR-based analysis of the viral relative copy number (C_N_) was performed. The value obtained from the HPV18 *wt* 3-day time point was set to 1, and the C_N_ values of other samples are expressed relative to this point. Panel D shows the effect of overexpression of E2C-1 on HPV18 *wt* replication, whereas panel E shows the effect of E2C-2.

Papillomaviruses encode a number of transcription regulators that control the levels of different viral transcripts and thus help to regulate viral genome copy number during amplification. The most studied and primal regulator is the E8^∧^E2 protein, which has been previously described in BPV1, in several Alpha HPVs (18, 31, 11, 16), in Mu-papillomavirus HPV 1 and in Beta-papillomavirus HPV5 [23], [32] [33], [34], [35], [36], [37], [38]. E8^∧^E2 consists of a short E8 product (11 amino acids) that is fused to the C-terminal part of the E2 protein, which contains DNA binding and dimerization domains. E8^∧^E2 downregulates HPV transcripts and is therefore a negative regulator of viral genome replication. We analyzed the transcripts initiated from P_3000_ and our analysis indicated that the P_3000_ promoter could generate two truncated forms of E2: E2C-1 and E2C-2 (80% of the RNA initiated from P_3000_, RNA U in Fig. 6). A similar promoter has been described previously for BPV1 and is responsible for the expression of E2-TR [33], [39], [40], [41]. Truncated E2 variants contain only C-terminal part of E2, similar to those that are described for BPV1 and HPV11 [42]. These truncated E2 proteins can act as repressors for HPV genome replication by competing with full-length E2 for E2-binding sites in the HPV18 genome or by forming heterodimers with full-length E2. E2C-1 is a spliced variant similar to E8^∧^E2 that consists of a short peptide starting from nt 3253–3255 in the E2 ORF (11 amino acids) that is spliced into the C-terminal part of E2. E2C-2 starts from position 3426–3428 and is an unspliced C-terminal E2 protein. We were curious wether the ATG-s for E2C1 and E2C-2 and splicing donor (SD) or splicing acceptor (SA) were conserved among Papillomaviruses. We analyzed 302 PV-s from PaVE database and found out that methionine used for E2C-2 is conserved in all α7 family HPVs (types 18, 39, 45, 59, 68, 70, 85 and 97) as well as in HPV67 (α9 family). It was also present in MfPV4, MfPV5, MfPV10 and PhPV1 Papillomaviruses. We next analyzed those PV-s that have methionine for E2C-2 for the presence of E2C-1. We found out that ATG for E2C-1 as well as splice donor (SD) and splice acceptor (SA) are conserved in α9 family. However the E2C-1 protein can exist only in case of HPV18 because other viruses have stop codon(s) between ATG and SD. To see if E2C-1 or E2C-2 have some kind of role in HPV18, we mutated ATG codons in E8, E2C-1 and E2C-2 alone and in combination and analyzed the initial amplification of these HPV18 genome mutants in U2OS cells. U2OS cells were electroporated with different miniplasmids of HPV18 genome mutants, and viral genome replication was measured 3 and 5 days after the transfection by Southern blot analyses (Fig. 7, panel A) and quantitative RT-PCR (Fig. 7, panel B, given values are expressed relative to the *wt* 3-day timepoint). Mutation in E8 ATG (E8-) resulted in an approximately 10-fold increase in replication signal compared to *wt*, as expected and as has been shown by others (Fig. 7 A, lanes 3 and 4; [23]). The E2C-1 mutation alone (E2C-1-) did not have any significant effect on genome replication compared to HPV18 *wt* replication (Fig. 7 A, lanes 15 and 16). Unexpectedly, the E2C-2 mutation (E2C-2-) decreased replication by at least 40% (Fig. 7 A, lanes 5 and 6), while double mutant in which both E2C proteins were knocked out (2E2C-) restored replication to the levels of *wt* (Fig. 7 A, lanes 7 and 8). Double mutants in which E8 and either E2C-1 or E2C-2 were mutated (E8-E2C1- and E8-E2C2-) exhibited 2.5-3-fold higher replication compared to the E8- genome, which indicates that at E2C variants regulate HPV18 genome replication but that E8^∧^E2 is a major repressor (Fig. 7 A, compare lanes 3 and 4 with 9/10 and 17/18). To further characterize the effects of E2C-1 and E2C-2 as the regulators of HPV18 genome replication, we constructed expression plasmids for these proteins under the CMV promoter. We developed a chicken polyclonal antibody against the C-terminal part of HPV18 E2 and used it in immunoprecipitation (IP)-Western analyses to evaluate the expression levels and molecular weights of E2, E2C1, E2C2 and E8^∧^E2. We easily detected all these proteins in the case of expression constructs, and their molecular weights corresponded to those predicted (Fig. 7, panel C). We next checked that the effects of the E2C-2 mutation in HPV18 initial amplification is not due to the additional mutation in the full-length E2 protein (M204V). We constructed an E2 expression vector p18E2_M204V. We complemented HPV18 E2 mutant genome defective in replication [17] with either p18E2 wt or M204V and saw no changes in the initial amplification of the mutant genome regardless of the mutation in the E2. Mutation M204V in the ORF of E2 protein did not alter the usage of nearby splice site 3434 as well (data not shown). To further characterize the effects of E2C1 and E2C2 on transient HPV18 replication, we transfected U2OS cells with the HPV18 genome with the addition of various concentrations of either E2C-1 or E2C-2 expression plasmids. An E8^∧^E2 expression vector was added as control. HPV18 replication was measured 3 and 4 days after the transfection with quantitative RT-PCR, and all the signals are expressed relative to *wt* replication at the 3-day timepoint. Concentration-dependent downregulation of viral genome replication clearly occurred, indicating that both E2C-1 (Fig. 7, panel D) and E2C-2 (Fig. 7, panel E) are negative regulators of HPV18 initial amplification.

### HPV18 E2C2 activates transcription in a concentration-dependent manner

**Figure 8:**
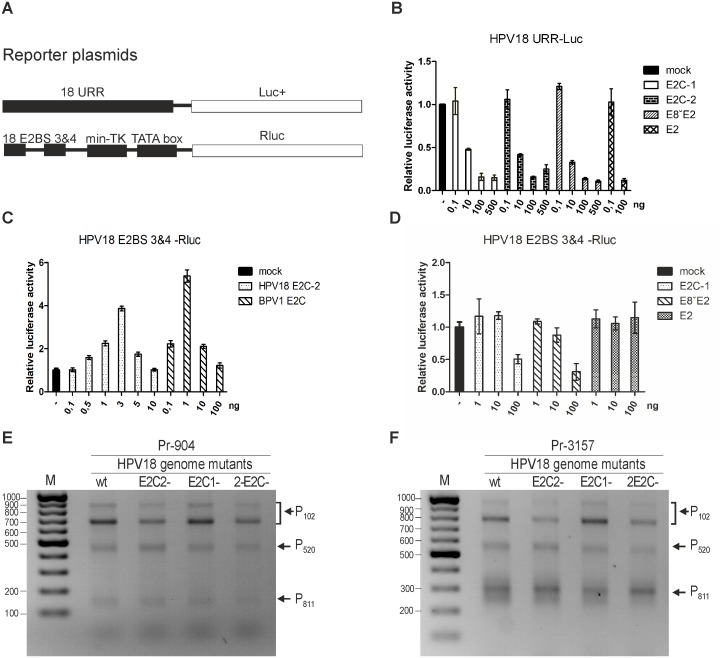
Transcriptional analyses of HPV18 E2 variants A: Schematic representations of two reporter plasmids. One reporter consists of full-length native HPV18 URR region acting as a promoter that drives the Firefly luciferase gene. The other reporter uses HPV18 E2-binding sites 3 and 4 as promoter. B: U2OS cells were transfected with the 18URR-Luc+ reporter plasmid and the non-specific pRL-TK reporter and together with different concentrations (0.1 ng, 10 ng, 100 ng and 500 ng) of the HPV18 E2C-1, E2C-2 and E8ˇE2 expression plasmids; 0.1 ng and 100 ng of the HPV18 E2 plasmid were added as controls. Twenty-four hours after the transfection, luciferase activities were measured and normalized to the Renilla values obtained from the pRL-TK reporter. These data indicate the effects of E2C-1, E2C-2, E8ˇE2 and E2 on the URR promoter activity relative to the reporter alone. Average values of two independent experiments are given. C: U2OS cells were transfected with the 18 E2BS 3&4-Luc+ reporter plasmid and non-specific pRL-TK reporter and together with different concentrations (0.1 ng, 0.5 ng, 1 ng, 3 ng, 5 ng or 10 ng) of HPV18 E2C-2 or 0.1 ng, 1 ng, 10 ng or 100 ng of the BPV1 E2C expression plasmids. Twenty-four hours after the transfection, luciferase activities were measured and normalized to the Renilla values obtained from the pRL-TK reporter. These data indicate the effects of HPV18 E2C-2 and BPV1 E2C on the E2BS 3&4 promoter activity relative to the reporter alone. Average values of three independent experiments are given. D: U2OS cells were transfected with the 18 E2BS 3&4-Luc+ reporter plasmid and non-specific pRL-TK reporter and together with different concentrations (1 ng, 10 ng and 100 ng) of the HPV18 E2C-1, E8ˇE2 and E2 expression plasmids. Twenty-four hours after the transfection, luciferase activities were measured and normalized to the Renilla values obtained from the pRL-TK reporter. These data indicate the effects of HPV18 E2C-1, E8ˇE2 and E2 on the E2BS 3&4 promoter activity relative to the reporter alone. Average values of two independent experiments are given. E and F: 5′ RACE analysis of HPV18 transcripts that were produced in U2OS cells during transient replication of *wt* or different mutant (E2C-2, E2C-1 and 2-E2C) genomes. HPV18-specific primers, Pr904, which binds to a site in the E7 ORF (panel E), and Pr3157 (panel F), which binds to a site in the E4 ORF, were used. Products representing the RNAs that are initiated from promoters P_102_, P_520_ and P_811_ are shown.

As shown in Fig. 7, panels A and B, viral genome replication decreased approximately 40% when E2C-2 was mutated in HPV18 *wt* genome. This reduction could indicate that besides being the repressor, E2C-2 can also activate replication by modifying HPV transcription from specific promoters. Experiments performed with BPV1 E2-TR, a protein similar to HPV18 E2C-2, show that C-terminal E2 proteins can activate transcription from different promoters [43]. Thus, we performed experiments to determine whether these proteins (E2C-1 or E2C-2) may carry an additional activation function in the case of HPV18. To evaluate transcription levels, we used two different reporter plasmids shown in Fig. 8, panel A. The first reporter has a full-length HPV18 upstream regulatory region (URR) as a promoter, and the second uses only HPV18 E2-binding sites 3 and 4, which drive Firefly luciferase expression. To measure the effects that different forms of E2 protein have on the transcription from the URR promoter, the transcription assay described in the Materials and Methods section was performed with various concentrations of E2C proteins. Full-length HPV18 E2 was added as a control because a strong inhibitory effect has been reported previously [23]. Both Firefly and Renilla luciferase activity levels were measured 24 hours after the transfection, and the data from the URR-Luc+ plasmid was normalized against the values obtained from pRL-Tk. The effects of the various HPV18 E2 variants are presented relative to the basal activity of the URR-Luc+ plasmid (mock). Because URR is a relatively strong promoter with several binding sites for cellular transcription factors, the binding of E2 inhibits transcription. As can be seen in Fig. 8, panel B, all of the truncated E2 proteins, E8E2C, E2C-1 and E2C-2, inhibited transcription from the URR promoter to the same extent in a concentration-specific manner compared to basal activity (mock). Next, we analyzed the effects of E2C-2 on transcription using a reporter plasmid which promoter region contains E2-binding sites 3 and 4, minimal-TK and TATA box, an approach that is more suitable due to lower baseline activity, thus allowing us to distinguish the effects of E2C-2 and cellular factors on transcription. We performed a transcription assay with various concentrations of the E2C-2 expression vector. A vector expressing BPV1 E2-TR was added as a positive control. We measured the luciferase activities and analyzed the data similarly to the full-length URR experiment. The results in Fig. 8, panel C indicate that HPV18 E2C-2 activates transcription at low concentrations up to 3-fold compared to the basal activity (mock). HPV18 E2C-2 is a much weaker activator than BPV1 E2-TR, which activated transcription up to 8-fold (Fig. 8, panel C). Next, we wanted to see how HPV18 E2C-1, E8^∧^E2 and full-length E2 modify the activity of E2BS 3&4 transcription. A similar experiment was performed, and we determined that neither of the proteins significantly altered the transcription from the promoter (Fig. 8, panel D) compared to its basal activity (mock). Because HPV18 E2C-2 can activate transcription, we were curious whether it alters some specific HPV18 promoter. Therefore, we transfected U2OS cells with HPV18 *wt* and with three different mutants: E2C-1, E2C-2 and 2-E2C. We conducted 5′ RACE analyses on the HPV18 transcripts during initial amplification of the HPV18 genome. Two different primers were used: Pr-904 binds to E7 ORF and Pr-3157 to E4 ORF. As can be observed in Fig. 8, panel E and F, in the case of the E2C-2 mutant or the double mutant 2-E2C, the levels of the RNAs initiated from promoter P102 were significantly weaker compared to those of the *wt* or the E2C-1 mutant. This result indicates that E2C-2 can activate P102 to some extent and explains the weaker replication that this mutant had (Fig. 8, panels A and B).

### HPV18 E8^∧^E1 has no significant role in transient replication

**Figure 9:**
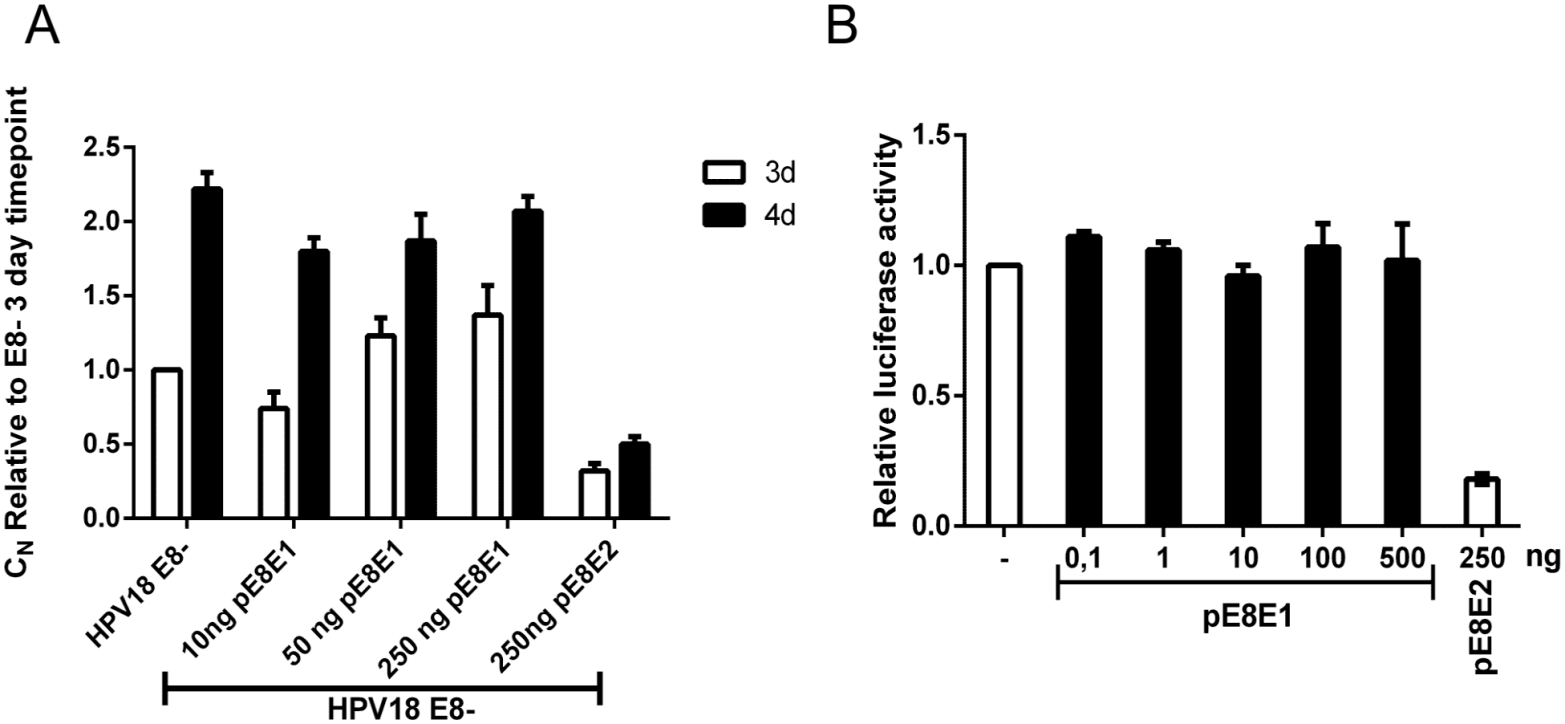
Analysis of E8ˇE1 overexpression on HPV18 genome replication and transcription. **A:** U2OS cells were transfected with 2 µg of the HPV18 E8 mutant minicircle alone or with different concentrations (10 ng, 50 ng and 250 ng) of the E8ˇE1 expression plasmid. The E8ˇE2 expression plasmid (250 ng) was added as a control. Genomic DNA was extracted 3 and 4 days after the transfection, and a qPCR-based analysis of the viral genome relative copy number (C_N_) was performed as described in the Materials and Methods section. The value obtained from the HPV18 E8 mutant 3-day timepoint was set to 1, and the CN values of the other samples are expressed relative to this point. The averages of the results of two experiments are presented with standard deviations. **B:** U2OS cells were transfected with different concentrations (0.1 ng, 1 ng, 10 ng, 100 ng and 500 ng) of E8ˇE1 or 250 ng of the E8ˇE2 expression plasmid together with the reporter plasmid URR-Luc containing the native HPV18 URR and a non-specific reporter pRL-TK. Twenty-four hours after the transfection, luciferase activities were measured and normalized to those for Renilla luciferase expression from the thymidine kinase promoter of the co-transfected plasmid pRL-TK. These data indicate the effect of E8ˇE1 on promoter activity relative to the reporter alone. The averages of the results of two experiments are presented with standard deviations.

When we analyzed transcripts containing E8 ORF, in addition to the known E8^∧^E2 transcript, we also identified the product E8^∧^E1 (RNA T in Fig. 6) described earlier in clinical samples and in HPV5 [38], [44], [45]. A protein product that can be encoded by E8^∧^E1 RNA, the E8 peptide is fused with the last 35 amino acids of the E1 protein. Because it is extremely difficult to specifically knock out this protein in the viral genome context, we constructed an expression plasmid for this protein driven by the CMV promoter. We transfected U2OS cells with HPV18 E8- genome together with various concentrations of pE8E1. HPV replication was analyzed 3 and 4 days after transfection by qPCR, and no changes in the viral copy number were observed (Fig. 9, panel A). All of the signals are given relative to the HPV18 E8-3 day timepoint. Next, we performed the transcription assay using a full-length HPV18 URR-Luc+ plasmid, as described in the Materials and Methods section. As can be observed in Fig. 9, panel B, expression of the HPV18 E8E1 protein did not alter the transcription levels. These results indicate that if E8E1 even exists at the protein level, it has no significant impact on HPV18 initial amplification.

## Discussion

The 5′ end mapping of the HPV18 transcripts in U2OS cells by a 5′ RACE assay revealed that the TSSs clustered into five distinct regions. These regions are referred to herein as P_102_, P_520_, P_811_, P_1193_ and P_3000_. Similar to observations from HPV18-infected raft cultures, the P_102_ region is most actively used to initiate the viral early transcripts during HPV18 replication in U2OS cells. The coding potentials of the P_102_-initiated transcripts (RNAs A-D, J and L-M in Fig. 6) include almost all of the viral early proteins, with the exception of E8^∧^E2. The P_102_ described herein includes TSSs that are distributed throughout a region from nt 7779 in the LCR to nt 201 in the E6 ORF. This region also includes the promoter P_55_, which was identified in infected raft cultures and in HeLa cells [18]. In U2OS cells, transcription initiation from nt 55 was rather rare (2% of analyzed clones). According to our data, the most prevalent TSSs in P_102_ were located at nt 102, 105 and 100, and two-thirds of the clones initiated from the TSSs at nt 102, 105, 100 or 97. These results are consistent with the data obtained from HPV18-infected raft cultures, in which the clones primarily initiated from TSSs at nt 102, 100 and 105 [18]. In U2OS cells, approximately 10% of the TSSs in P_102_ were located in the region upstream of nt 55. However, this phenomenon is not unique to U2OS cells. For example, in addition to the main TSSs around the start codon of the E6 ORF, initiation sites located approximately 100, 115, 210 and 350 nt upstream (approximately nt 7615-5) were detected in HPV18-positive cervical cell lines [26].

The region P_520_ located within the E6 ORF is quite narrow compared to other HPV18 promoter regions. The TSSs of P_520_ are located primarily in a 65-bp region (nt 479 to 544), with the exception of a TSS that was detected at nt 414 in subclone #1.13 cells. This region has lower activity than P_102_. All RNAs that initiated from P_520_, which were mostly RNA E and rarely K or N (Fig. 6), were spliced at the donor site at nt 929, and the coding potentials of these transcripts include the E7, E1^∧^E4 and E5 proteins. Transcription initiation within the E6 ORF was observed previously in HPV18 and HPV16 ([18], [46]).

The P_811_ region was defined previously as the viral late promoter for the expression of L1 and L2 mRNAs from scattered TSSs that extend over a region from nt 674 to 840, most frequently at nt 811 [18]. In U2OS cells, TSSs of clones from this region indicated a preference for the TSS at nt 829 in a range from nt 689 to 903, with the exception of a clone that initiated from nt 641. Here, we also show that P_811_ can be used to initiate transcripts that encode the viral early genes. In the transient replications, we did not detect the use of the late PAS that is needed for the generation of viral late transcripts. However, the P_811_ transcripts were detected at 22 and 71 h after HPV18 genome transfection, although these transcripts were present in lower amounts compared to those that initiated from P_520_ and P_102_ (Fig. 5, panels D and E). These transcripts have coding potentials for the E1^∧^E4 and E5 proteins (RNAs F and O, Fig. 6) and, when spliced at nt 2779, for the E2 protein (RNA S, Fig. 6). Promoter activity in the E7 ORF was demonstrated previously in HPV16 [47], [48] and HPV31 [49].

Consistent with observations by Wang et al., it appears that P_1193_ is used for the production of mRNAs that encode the E8^∧^E2 protein, which has been identified as a negative regulator of viral replication in HPV types 18, 16 and 31 [23], [37], [50]. The E8^∧^E2-encoding transcript (RNA G, Fig. 6) was detected during transient replication in U2OS cells and in subclone #1.13 cells. The RNA initiated from P_1193_ and spliced at acceptor site nt 2779 can encode the full-length E2 protein (RNA T, Fig. 6). This transcript was present in the transient replication samples at a much lower level (less than 5% of product 1; Fig. 5A).

Region P_3000_ within the E2 ORF has been described previously for HPV18 in vitro and in Hela cells [20] but the transcripts initiated from this promoter have not been analyzed. Besides HPV18, the promoter activity inside of the E2 ORF has been observed for HPV31 [51]. However, herein, the HPV18 transcription initiation activity from this region was detected in both transiently transfected cells and subclone #1.13 cells. Three types of transcripts were expressed from P_3000_: those spliced at nt 3165/3434 or 3165/3465 (RNAs H and R, Fig. 6); those spliced at nt 3284/3434 (RNA I, Fig. 6); and those remaining unspliced (RNA U, Fig. 6). Although not previously described in HPVs, in BPV1, it is known that a promoter within the E2 ORF is used for the generation of the E2C (E2-TR) repressor protein, which consists of the C-terminal part of the E2 activation domain, the E2 hinge and the DBD [39], [52]. We identified the putative ATG start codons for the HPV18 E2C proteins at nt 3253 for E2C1 (RNA I, Fig. 6) and 3426 for E2C2 (RNA U, Fig. 6), downstream of the P_3000_ TSSs, and hypothesized that transcripts generated from P_3000_ might be used to produce the HPV18 E2C proteins. Both E2C1 and E2C2, like E8^∧^E2, might act as inhibitors of E2-mediated functions via competitive binding to E2-binding sites in the viral genome and by the formation of heterodimers with full-length E2 [53], [54]. Among other activities, the E8^∧^E2 proteins are thought to regulate viral replication and copy number [23], [37], [50], [55]. Mutational analysis indicated that abrogation of the putative E2C1 start codon alone had no effect on the viral genome copy number. In combination with a mutation in the E8^∧^E2 start codon (E8^−^), the effect on copy number was clearly detectable because the double mutant had a considerably higher copy number than the E8^−^ mutation alone. In contrast, the abrogation of the E2C2 start codon resulted in an approximately 40% decrease in the transient replication of HPV18 in U2OS cells. However, the double mutant bearing both E8^∧^E2 and E2C2 mutations exhibited considerably higher replication levels than the E8- mutant. To further characterize the roles of E2C1, E2C2 and E8^∧^E2, we engineered expression vectors for these proteins. IP-Western analysis using a chicken polyclonal antibody against the C-terminal part of HPV18 E2 was used to determine that those proteins were actually synthesized and that their molecular weights (MWs) corresponded to the predicted weights. We also tried to detect the proteins from transiently replicating HPV18 genomes; however, the protein levels were too low to detect (data not shown). To further characterize the roles of E2C1, E2C2 and E8^∧^E2 in transient replication, we showed that overexpression of these proteins dramatically decreases viral replication, indicating that all three can act as repressors for HPV18. The results indicate that E8^∧^E2 represents a major repressor, and at least in transient replication, copy number control can be achieved mostly by this protein. In comparison, E2C1 and E2C2 have minor effects. However, it is possible that E2C1 and E2C2 have more crucial roles in other viral life cycle stages, such as the conversion from initial amplification to stable maintenance replication, or that they have other functions that are not directly associated with genome copy number control. BPV1 E2-TR acts in combination with cellular components as a transcriptional activator that is capable of modulating BPV early gene expression in a manner distinct from E2 and E2^∧^E8 [43]. Because E2C2 mutation alone resulted in a decrease in viral replication, we hypothesized that this effect might also be the case for HPV18. We showed that, in certain conditions, E2C2, but not E2C1, E8^∧^E2 or full-length E2, can actually activate transcription by binding to the HPV18 E2-binding sites 3 and 4. Furthermore, we conducted 5′ RACE analyses on the transient replication of the HPV18 E2C2 mutant and observed a clear downregulation of P_102_, indicating that apart for E2, E2C1 and E8^∧^E2, E2C2 is also used to control the transcriptional activity during HPV18 transient replication in U2OS cells. Taken together, these results indicate that HPV18 uses four genome copy number regulators during transient replication: E2, the major repressor E8^∧^E2, the minor repressor E2C1 and the regulator E2C2. During the transcription analyses of HPV18 transient replication, we identified an mRNA that can be used to translate the E8^∧^E1 protein, described previously for HPV5, HPV11 and HPV47 [38], [44], [45]. We used an expression vector for this fusion protein to characterize the effects of E8^∧^E1 in the transient replication of HPV18. However, we were unable to show any effect on the HPV18 copy number, and this protein does not alter transcription from the HPV18 URR region. These results indicate that even if the specific mRNA is used to translate E8^∧^E1, it has no significant role during the first replication stage of the HPV18 in U2OS cells.

The existence of multiple TSSs located at some distance from one another is common to HPV promoter regions defined herein and by others [18], [26], [47]. However, TSSs in regions P_102_, P_520_, P_811_ and P_1193_ mostly contain the purine A at position +1, and the motif around the TSS is often similar to the human consensus sequence Y**A**NW (Table 1) [28]. Moreover, with the exception of the TSS from P_3000_, the most frequently used TSS in each region includes this consensus motif.

The splicing sites in the viral early region in U2OS cells that we detected were generally the same as those that were previously identified in raft cultures during productive infections [18] or in clinical samples [31]. In addition to these sites, splicing at nt 3440 and 3284 (discussed above) was observed in U2OS cells. Differences in the usage of the alternative splicing acceptor sites at nt 3434, 3440 and 3465 indicated a strong preference for the splicing donor site at nt 3434. This preference may reflect a regulatory mechanism of viral gene expression via alternative splicing because the use of the splicing acceptor site at nt 3465 instead of those at nt 3434 or nt 3440 results in a change of the reading frame.

Most of the transcripts identified in U2OS cells were also detected in a human keratinocyte raft culture that was productively infected with HPV18. In addition, the PASs used for early and late transcripts in raft tissues [18] are identical to those identified in U2OS cells. These observations confirm that the gene expression profile of HPV18 in U2OS cells is similar to that in undifferentiated keratinocytes, the natural environment for HPV. Similar observations have been made concerning HPV5 and HPV11 genome replication in U2OS cells [38] (Isok-Paas et al., manuscript in preparation). Thus, the observed HPV genome replication in U2OS cells is not simply based on origin-containing plasmid propagation when E1 and E2 are expressed either from the same plasmid or in trans. Rather, it is the reconstitution of many events that are associated with natural viral infections when the viral factors are functionally expressed at the physiologically relevant levels. Events that are reminiscent of the late-phase viral life cycle, include amplification of the viral genome and increased cytokeratin K10 expression, have been observed previously in HPV-infected U2OS cells that were cultivated as confluent cultures and fed regularly [17].

Additionally, we demonstrated the induction of mRNAs that contain the E1 ORF sequence in U2OS cells maintained in conditions that promote HPV18 genome amplification, which is consistent with data published in ref. [17]. During transient replication and in subclone #1.13 cells maintained in conditions to ensure the stable maintenance of the HPV18 episomal genome, E1-encoding transcripts were expressed only from the P_102_ region. However, the increase in E1 ORF transcripts that is associated with the amplificational mode of replication was achieved by increases in the quantities of both transcripts expressed from the promoter region P_102_ and additional E1-encoding transcripts from the promoter regions P_520_, P_811_ and P_1193_ during viral genome amplification. Of these transcripts, mRNAs from P_520_ and P_811_ permit production of the E1 protein. Thus, the data indicate that other promoters, such as P_520_ and P_811_ (which was which were previously associated with late gene expression [18]), may be involved in the production of E1-encoding transcripts during amplificational replication of the viral genome. Whether these transcripts are polyadenylated at the early or late PAS is not clear. Interestingly, the use of the late PAS was detected only in subclone #1.13 cells that were maintained in conditions to induce viral genome amplification. The nature and coding potential of the viral transcripts that were polyadenylated at the late PAS are currently under investigation. Furthermore, previously, it was shown that the HPV18 E1 protein is translated from a polycistronic mRNA consisting of the coding sequences for the E6, E7, and E1 proteins [56] and that a frameshift mutation in the E7 ORF increases E1 expression from the HPV18 genome [24]. The E1 ORF transcripts that initiated from P_520_ and P_811_ contained very little of the E6 ORF (when initiated from P_520_) or did not contain any of the E6 ORF and instead contained part of the E7 ORF (when initiated from P_811_).

Collectively, the data provided herein confirm the usefulness of the U2OS system for studies of the fundamental properties of papillomaviruses. Furthermore, we believe that the similarities of the transcription maps of HPV18 from U2OS cells and in raft cultures indicate that the U2OS system is an excellent platform for applications related to the development of antiviral compounds. In particular, U2OS cells, which support the replication of many different HPVs, could be utilized to develop cell-based assays for the high-throughput screening of anti-HPV compounds.
